# The Drug Treatment of Inebriety

**Published:** 1906-03-24

**Authors:** 


### THE DRUG TREATMENT OF INEBRIETY.

Dr. Jos. S. Bolton,[1] writing of his own experience during the last twelve months in the drug treatment of inebriates, claims a record of distinct progress, though he declines to announce any "cure," as he considers this term should only be applied to an inebriate who, after treatment, has been an abstainer for at least two years. The following are some practical points to which Dr. Bolton draws attention. At first he used strychnine and atropine in what proved to be unnecessarily large doses, and the resulting dryness of the throat and difficulty of vision caused some patients to discontinue the treatment. He now limits the dose to one minim of solution of sulphate of atropine and four minims of the solution of hydrochloride of strychnine. This he gives by subcutaneous injection twice a day for a month, then once a day for a fortnight, and again every second day for another fortnight. Afterwards the patient is seen at least once a week, and if at any time there is a complaint of mental depression or a craving for drink, the injections are resumed for a few days until the danger is passed. The patients, with one exception, were all treated as out-patients. Most of them took for the first few days a mixture containing capsicum and cinchona, but it is not necessary to continue this after the appetite has returned and the "low feeling" at the stomach has passed away. Even though relapses occurred in several instances, it was found possible to induce the patient to return for further treatment, and this, in more than one case, with a satisfactory result.

[1] Brit. Med. Jour., March 3, 1906.

